# Working memory training in healthy young adults: Support for the null from a randomized comparison to active and passive control groups

**DOI:** 10.1371/journal.pone.0177707

**Published:** 2017-05-30

**Authors:** Cameron M. Clark, Linette Lawlor-Savage, Vina M. Goghari

**Affiliations:** 1 Department of Physiology and Pharmacology, University of Calgary, Calgary, Alberta, Canada; 2 Cumming School of Medicine, University of Calgary, Calgary, Alberta, Canada; 3 Hotchkiss Brain Institute, Calgary, Alberta, Canada; 4 Department of Psychology, University of Calgary, Calgary, Alberta, Canada; 5 Department of Psychology & Graduate Department of Psychological Clinical Science, University of Toronto Scarborough, Scarborough, Ontario, Canada; Tilburg University, NETHERLANDS

## Abstract

Training of working memory as a method of increasing working memory capacity and fluid intelligence has received much attention in recent years. This burgeoning field remains highly controversial with empirically-backed disagreements at all levels of evidence, including individual studies, systematic reviews, and even meta-analyses. The current study investigated the effect of a randomized six week online working memory intervention on untrained cognitive abilities in a community-recruited sample of healthy young adults, in relation to both a processing speed training active control condition, as well as a no-contact control condition. Results of traditional null hypothesis significance testing, as well as Bayesian factor analyses, revealed support for the null hypothesis across all cognitive tests administered before and after training. Importantly, all three groups were similar at pre-training for a variety of individual variables purported to moderate transfer of training to fluid intelligence, including personality traits, motivation to train, and expectations of cognitive improvement from training. Because these results are consistent with experimental trials of equal or greater methodological rigor, we suggest that future research re-focus on: 1) other promising interventions known to increase memory performance in healthy young adults, and; 2) examining sub-populations or alternative populations in which working memory training may be efficacious.

## Introduction

Working memory (WM) is the set of cognitive processes that work to maintain and manipulate task-relevant information during cognitive task performance, while also preventing interference from task-irrelevant information. In this sense, WM is an interplay between attention and memory that allows for temporary access to intermediate mental representations needed for more complex cognition. By briefly preserving task-relevant information, and facilitating manipulation of it, WM allows us to act outside the bounds of the immediate moment, and to coordinate complex and goal-directed behaviours [1–2]. As such, WM is a core cognitive ability in humans, and underlies performance on virtually all complex cognitive tasks, both within and beyond the laboratory. People differ in terms of how much information they can store in WM, and also in how readily they can store this information in the face of distraction [3]. While the absolute value of these inter-individual differences in WM capacity may in fact be quite small (e.g. 2 versus 6 items for low- and high-ability individuals respectively; [4]), these differences have been found to be highly predictive of performance on a wide variety of cognitively demanding tasks, including: reading comprehension, language abilities, mathematics, reasoning, problem solving, and also overall academic performance [5–6].

In addition to driving variation in scholastic achievement and educational success, WM ability has also been found to be highly related to the ability to acquire knowledge, to learn new skills, and also to the construct of ‘fluid intelligence’ more broadly [7]. In the theory of Cattell [8], ‘fluid intelligence’ (*Gf*) is the ability to adapt our reasoning abilities to solve novel cognitive problems. In contrast, ‘crystallized intelligence’ (*Gc*) draws heavily upon previously learned culturally-rooted knowledge acquired from education and previous experience [9–11]. Fluid intelligence and WM are highly related psychological constructs. Working memory capacity has been established as one of the best predictors of general intelligence [12], and investigations of the strength of the relationship between WM and *Gf* in particular have indicated moderate correlations with coefficients in the .3 to .9 range [13–14]. Similarly, Martinez and colleagues [15] describe WM capacity and *Gf* as almost isomorphic, and Chuderski [16] noted latent factors of the two constructs being statistically indistinguishable when time limits were imposed on test takers. General intelligence itself, perhaps unsurprisingly, has been linked to a wide variety of important life outcomes, including academic success [17–18], job performance [19], income [20–21], health [22–23], morbidity [24], mortality [24–25], and crime [17].

Given the strong relationship between WM and *Gf*, and the wide range of social, educational, and occupational outcomes to which they are positively correlated, it is no surprise that recent research has intensely focused on developing interventions to increase them via training [6, 26]. Halford, Cowan, & Andrews [27] posited a model by which facilitation of one cognitive ability might then transfer to a different *untrained* ability. Specifically, they argued that *Gf* and WM are related in that both share a common capacity constraint due to a shared demand for attention in respective reasoning or memory tasks. Under this model, while a common capacity limit may be expressed in terms of the number of items a person is able to hold in WM, the same capacity limitation may be expressed in terms of the number of interrelations amongst elements a person is able to maintain during a reasoning task indicative of *Gf* ability. The general idea is that if working memory capacity could be increased, even just marginally by training, performance on other cognitive abilities that are strongly related to it (like *Gf*) ought to thereby be augmented as well.

Jaeggi and colleagues [28] put this theory to the test, and found significant facilitation of performance on tests of *Gf* following WM training in a healthy young adult population. Empirical study on WM training and its effects on *Gf* has greatly intensified since the publication of Jaeggi et al.’s [28] initial positive findings (see [29–35]). However, although many studies have found strong and durable effects (over several months) for near-transfer (i.e. facilitation of WM capacity by WM training) of WM abilities, examples of far-transfer (i.e. facilitation of untrained abilities by WM training) to *Gf* have been more elusive, as well as generally weaker and less durable when they have been found (see [26, 36–45]). Rather, to this point there exists a striking lack of consensus in the literature about whether or not training on WM tasks generalizes to *Gf*, and secondly, the specific methodology by which these claims ought to be tested. The topic remains highly controversial and has spurred a variety of conflicting reviews [46–52], meta-analyses [53–57] meta-analytic rebuttals [58], meta-analytic counter-rebuttals [59], and even further meta-analytic rejoinders [53] on the basis of existing trials. The resulting literature on the efficacy of WM training is what Urbánek and Marček [60] have candidly called “reliably ambiguous” in terms of efficacy. Unfortunately the cumulative effect of this literature has been to jointly obfuscate the ostensibly simple question that each individual experiment, review, and meta-analysis has sought to clarify: “Does working memory training work?”

Subsequent investigations and reviews have addressed a variety of methodological shortcomings thought to account for the early positive findings in the field (see [61]), however, new and more specific methodological qualms have since arisen in the literature in an attempt to further homogenize study design, and encourage the search for additional unmeasured or uncontrolled variables which may account for significant variance in extant WM training trials. The search for these variables can generally be divided into two main types: 1) those relating to individual differences amongst WM training participants themselves; and 2) those relating to WM training trial design and execution.

Relating to individual differences amongst participants, Urbánek and Marček [60] rightly point out, that from a conceptual point of view, the reliably ambiguous nature of the WM training literature may be the result of an (as of yet unmeasured) independent, randomly distributed factor in participants. For example, Chein and Morrison [29] noted that no study up to that date had accounted for the potential effects of motivation, commitment, or training task difficulty across experimental and control conditions. Jaeggi and colleagues [6] echoed these concerns, and further suggested that individual differences in personality factors, pre-existing ability, and intrinsic versus extrinsic motivational factors need to be considered when assessing WM training and transfer.

Relating to WM training trial design and execution, Redick and colleagues [52] discuss several methodological issues ubiquitous in the WM training literature as a type of ‘best practices guide’ to study design. Firstly, they advocate for the use of sensible active control groups over simple no-contact control groups. When compared to no-contact control groups alone, active training groups may benefit from a number of advantages related to the placebo or Hawthorne effects. Secondly, they stress the importance of adequate sample sizes, and recommend at least 20 participants per group, following Simmons, Nelson, & Simonsohn [62]. Small sample sizes are unfortunately common in the working memory training literature likely due to the time and cost associated with the intervention, and can produce inflated effect sizes. Third, if facilitation of *Gf* by WM training is to occur by increasing the capacity of WM (as per Halford et al.’s model [27]), evidence of this intermediate step should also be demonstrated along with evidence of the far-transfer by a separate task from the training task itself. Fourth, the pattern of results supporting the transfer effect should be ‘sensible’. That is, further than simply achieving a significant group by time interaction effect, this result should be achieved within the context of relatively equal group performance at pre-training testing, and divergent performance at post-training in favour of the active training group (see Redick [63] for examples of studies with ‘non-sensible patterns of significant results). Finally, Redick and colleagues [52] advocate for including more than one outcome measure for far-transfer to *Gf* which can then be used to form a composite or latent variable for subsequent analyses.

Meta-analytic work [53–55, 57, 64] has pointed to a number of potentially moderating factors of WM training trial success or failure, including type of cognitive training (n-back training versus other types), participant age (younger versus older), participant status (learning disabled versus impaired WM versus normal functioning), training dose (less versus more), randomization (randomized versus nonrandomized), type of control group (treated versus untreated), geographic location (United States versus international populations), remuneration for participation (more versus less), and publication type (theses, dissertations, and conference posters versus journal articles, book chapters, and peer-reviewed conference proceedings). Unfortunately, as alluded to above, the authors of these meta-analytic reviews have disagreed about the appropriate methods for conducting a meta-analytic review of WM training, which have led them to opposite conclusions about the efficacy of WM training overall.

Melby-Lervåg et al.’s latest meta-analytic review [53] addressed several shortcomings in previous meta-analytic work in examining 87 publications with 145 separate experimental comparisons of WM training groups versus treated control groups. The authors did find a significant effect of cognitive training for nonverbal ability in adults (*g* = 0.10; *p* < .05), and for n-back training specifically (*g* = 0.15; *p* = .02) in studies using treated control (effect sizes jump to 0.20 and 0.26 respectively when examining studies comparing to untreated controls). However, closer examination of the studies that contributed to this significant positive effect size were found to suffer from several of the methodological shortcomings described by Redick and colleagues [52]. For example, the five largest effect sizes were arrived at with sample sizes of less than 20 per group, and employed only a single outcome measure of nonverbal ability. More troublingly, four of these five largest effect sizes evinced substantial unexplained decreases in outcome measure scores for the control group, which were in fact larger than the increases observed in the training groups. These nonsensical (or at least conceptually counterintuitive) ‘crossover patterns’ of training effect [63, 65] artificially inflate the effect sizes for individual comparisons, as well as for averaged estimates in meta-analyses. Melby-Lervåg and colleagues [53] additionally note that the effect size of n-back training on nonverbal ability drops below significance when only the most problematic of these five studies is removed from the analysis. Perhaps most troublingly of all, observed gains in nonverbal ability were not found to be significantly related to increases in WM abilities themselves, thereby casting doubt on the proposed mechanism of far-transfer discussed by Halford et al. [27]. Overall, Melby-Lervåg and colleagues [53] conclude that while there is convincing evidence of large improvements on tasks similar to those utilized by WM training (i.e. near-transfer, and ‘intermediate transfer’ to visual and verbal WM), there are no convincing effects of far-transfer of WM training to constructs such as nonverbal ability, verbal ability, reading comprehension or arithmetic that could not otherwise be explained by methodological shortcomings. Importantly, and contrary to the suggestions in the literature regarding potential effects of individual differences, moderator analyses revealed no evidence of moderation effects for nonverbal ability (e.g. participant age, status, training dose, training type etc.) aside from significantly higher effect sizes for studies utilizing untreated controls versus those implementing treated control groups. Crucially, Melby-Lervåg and colleagues [53] demonstrated the effect of adequate sample size and control group treatment by pooling effect sizes for studies falling into the four resulting permutations of study design (i.e. ≥ 20 participants and treated controls, ≥ 20 participants and untreated controls, < 20 participants and treated controls; < 20 participants and untreated controls). Average effect sizes in each of these conditions showed significant effects for far-transfer of WM training to nonverbal ability, except for the most conservative and robust experimental design (i.e. ≥ 20 participants and a treated control group), which showed an average effect size close to zero (g = 0.01).

Given the rapidly expanding and evolving field of WM training, the present study seeks to address whether or not the pattern of far-transfer of ability from WM capacity to *Gf* can be replicated while addressing several of the methodological shortcomings ubiquitous to the current literature. The most up to date meta-analytic review of the field at the time of planning the current study was that of Melby-Lervåg and Hulme [54], which included results from 30 comparisons from 23 studies carried out between 2002 and 2011. While more recent reviews (discussed above) have become increasingly pessimistic about true effects of WM training, they also have the benefit of drawing from a pool of experimental investigations almost five times as large as that of Melby-Lervåg and Hulme’s initial work in 2013, just four years later (recall that Melby-Lervåg et al.’s latest meta-analytic review [53] includes 145 comparisons from 87 separate studies). Thus, while the most up to date reviews tend to support the null hypothesis, earlier reviews were somewhat more optimistic–and particularly so for n-back training in young adults transferring to nonverbal abilities.

On the basis of these early initial estimates of effect size in the literature, we hypothesized that: 1) WM trained participants would demonstrate increased task performance on the training tasks themselves, 2) as well as increased WM capacity (i.e. near-transfer), compared to our treated and untreated control comparison groups. We additionally hypothesized that: 3) participants in the WM training group would exhibit far-transfer of ability to untrained tasks via increased test scores on measures of *Gf* compared to the treated and untreated control groups.

## Materials and methods

### Participants and recruitment

**Fig 1:**
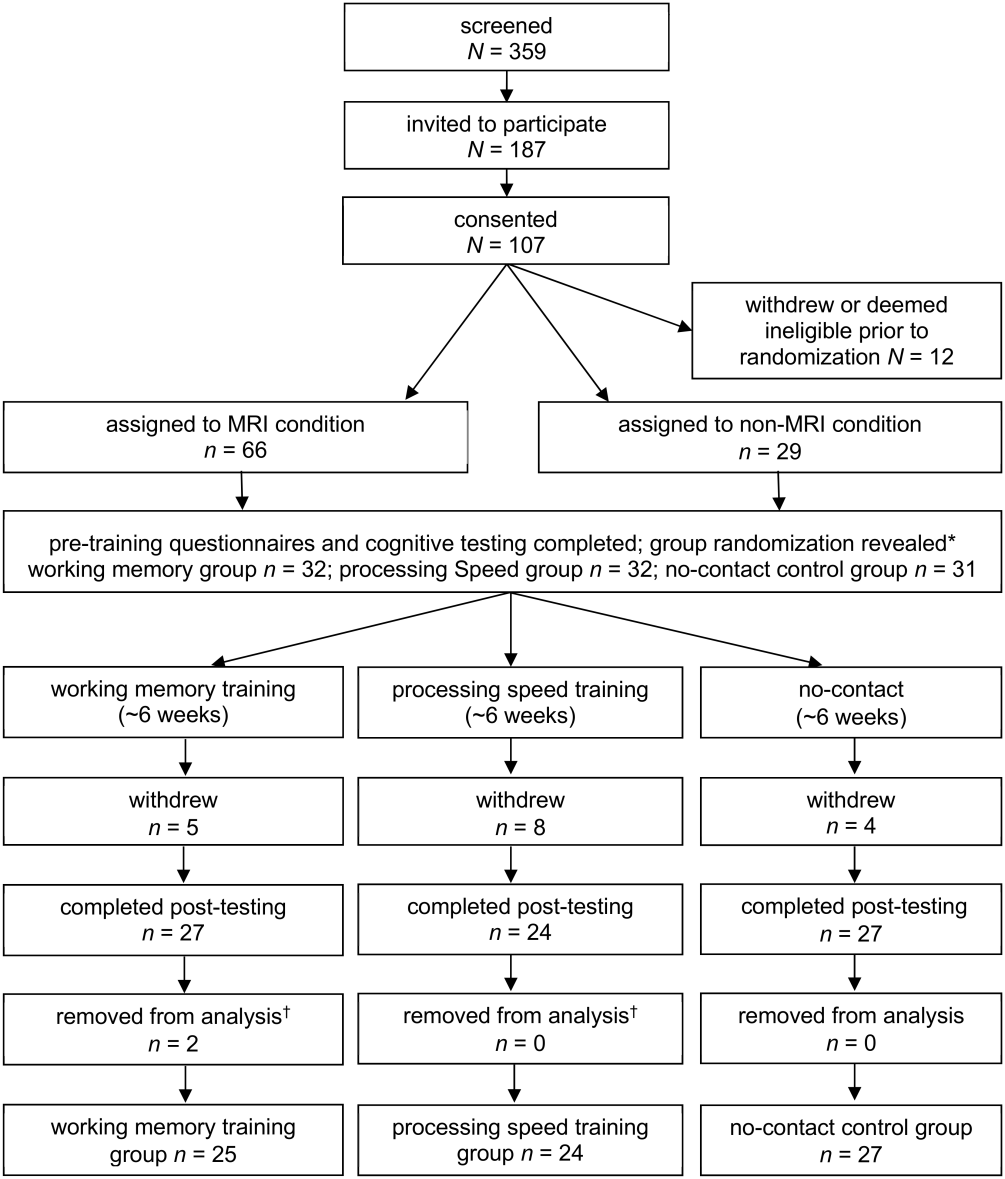
Flow chart of study design. *Two participants in MRI conditions were reassigned to the no-contact control group after being unable to tolerate MRI scanning. ^†^Participants removed from analysis due to training contamination, low training dosage, or data acquisition issues.

A total of 359 healthy adults responded to printed advertisements distributed throughout the community as part of a larger neuroimaging WM training trial. All MRI procedures and results are discussed in two forthcoming manuscripts by these authors. The main text of the printed advertisements read: “Participants Needed: Brain Training Neuroimaging Study. For more information visit our website braintrainingstudy.ca” (see S1 Fig for the poster itself). Potential participants completed online screening measures at braintrainingstudy.ca which inquired about study exclusion criteria, including: 1) age less than 18, or greater than 40; 2) left-handedness; 3) history of traumatic brain injury or other neurological condition causing sensory or motor impairment; 4) self-reported presence of Axis I mental illness; 5) less than normal or corrected-to-normal visual acuity; 6) MRI contra-indications; 7) insufficient access to a computer and high-speed internet; and 8) recent or previous use of the n-back training task or other online cognitive training paradigms. Of the 359 potential participants who completed the screening questionnaires, 187 were invited to participate in the study, and a total of 76 participants were ultimately included in the analyses. See Fig 1 for a flow chart depicting the recruitment, randomization, and exclusion process. Participants were compensated $20 per cognitive testing session, and $20 per MRI session, totalling $80 for the four appointments attended by participants randomly assigned to the MRI conditions, and $40 for the two appointments attended by those assigned to the no-contact control condition. Written consent was obtained from all participants, and ethics approval was obtained from the University of Calgary’s Conjoint Health Research Ethics Board (CHREB).

### Procedure

Following initial recruitment and screening, participants were randomized to one of three groups: a WM training group (*n* = 25), a processing speed (PS) active control group (*n* = 24), or a no-contact control group (*n* = 27). PS training was chosen as an active control condition on the basis of its association with robust improvements on measures of processing speed, but not measures of WM, inhibition, or nonverbal reasoning [66]. Thus, preliminary evidence suggests that PS training may offer a viable active control condition to WM training by holding constant the level of effort, motivation, and interaction with computers and researchers, while impacting relatively orthogonal behavioral skillsets [66–67].

Participants were blinded to group randomization with respect to the WM and PS training groups. However, because assignment to the no-contact control group entailed not undergoing MRI scanning sessions, and not completing online training, participants in this group were aware of their group assignment. Efforts were made to blind experimenters to group assignment, though the distinction between training groups versus no-contact control was similarly difficult to blind because of the difference in the number of scheduled appointments (i.e. two additional MRI appointments for participants in the WM and PS training groups). In this sense, the experimenters cannot be considered to have been truly blind to group assignment. Importantly however, the experimenters were typically unable to distinguish between those in the WM training versus PS active control groups when meeting them for MRI or cognitive testing appointments. Following group assignment, participants in the WM and PS training groups underwent their initial MRI session, and then completed initial cognitive testing appointments on a separate day shortly thereafter. They were then given login access to Lumosity.com [68] where they were asked to complete specially designed online training programs targeting either WM or PS cognitive processes. Participants were asked to allow 20–30 minutes of training per day, for five out of seven days per week, for six weeks. Progress in training was monitored online for each participant, and individuals were removed from the study if they did not complete at least 20 of the assigned 30 days of cognitive training over the six week training period. Participants were also removed from the study if they erroneously accessed Lumosity training games outside of those prescribed by their training program. However, we were unable to track whether or not participants accessed other Lumosity games using different login credentials, or other ‘brain training’ programs entirely. Encouragement emails were sent to participants on a weekly basis in order to facilitate compliance with the prescribed training regimen. Following training, participants in the WM training and PS active control groups underwent a second cognitive assessment. Participants in the no-contact control group simply completed cognitive testing on two occasions, approximately six weeks apart.

### Cognitive testing and behavioral measures

Cognitive testing included split-half subtests from the Wechsler Adult Intelligence Scale—Fourth Edition (WAIS-IV) [69], Raven’s Advanced Progressive Matrices (RAPM) [70–71], and two parallel forms of Cattell’s Culture Fair Test (CCFT) [72–73]. Parallel forms (i.e. split halves) of these cognitive measures were not randomized across pre- versus post-training assessments, though order of administration was pseudorandomized. Thus, participants in all groups completed odd numbered items (and form A of the CCFT) before training, and even numbered items after (and form B of the CCFT), in the same pseudorandomized order across both testing sessions. Computerized administrations of the Automated Operation Span Task (AOSPAN) [74], and a Spatial Delayed Response Task (SDRT) [75] were also administered both before and after training. Cognitive assessments were completed by PhD-level graduate students with specific training in neuropsychological assessment, or undergraduate volunteers trained and supervised by the graduate students. Assessment sessions were typically 100 to 120 minutes in duration.

#### Wechsler Adult Intelligence Scale—Fourth Edition (WAIS-IV)

Eight of the 10 core subtests of the WAIS-IV were administered in order to allow calculation of all four composite indices of intelligence assessed by the WAIS-IV: Verbal Comprehension Index (VCI), Perceptual Reasoning Index (PRI), Working Memory Index (WMI), and Processing Speed Index (PSI). These included ‘Vocabulary’, ‘Similarities’, ‘Block Design’, ‘Matrix Reasoning’, ‘Digit Span’, ‘Arithmetic’, ‘Symbol Search’, and ‘Coding’. All subtests were split in half for pre- versus post-training comparison, with the exception of Digit Span, Symbol Search, and Coding, which were administered in their entirety before and after training. Discontinue rules for split-half subtests were halved and rounded up where necessary.

#### Raven’s Advanced Progressive Matrices (RAPM)

RAPM [70–71] is a reliable and well validated paper and pencil test of general cognitive ability. Participants are asked to examine a matrix pattern with a missing piece, and select the correct answer from eight possible answers. RAPM is published in two sets: Set-I which contains 12 screener and/or practice items and has a five minute time limit, and Set-II which contains 36 items and has a 40-minute time limit. Due to the split-half protocol, at each cognitive testing session participants completed six practice items within five minutes, followed by as many of the 18 test items as they could within a 20-minute time limit.

#### Cattell’s Culture Fair Test (CCFT)

CCFT [72–73] is a test of general reasoning and cognitive abilities that was designed specifically to reduce emphasis placed on linguistic abilities and general store of culturally specific knowledge in traditional tests of intelligence. The test contains two equivalent forms, each consisting of four subtests: series, analogies, matrices, and classification, and thus provides a more varied assessment of general cognitive functioning beyond matrix reasoning ability as assessed in isolation by the RAPM [76].

#### Automated Operation Span Task (AOSPAN)

The AOSPAN task [74] is a complex measure of WM which requires participants to remember the sequential ordering of presented stimuli while carrying out simple mathematic problems as a distraction. The dependent variable of interest is the number of correctly recalled letters in each trial.

#### Spatial Delayed Response Task (SDRT)

The SDRT [75] assesses visuospatial working memory by briefly presenting participants with a series circles on a computer screen, and requires that they determine whether a second set of circles is the same after a two second delay. A second condition asks participants to determine whether the second set of circles is the same as the first set, but flipped about the horizontal midline of the presentation space. Across a variety of difficulties (1, 3, 5, or 7 circles presented), the variable of interest is the total number of correct trials for both with- and without-manipulation (i.e., flipped) conditions.

#### Additional behavioural measures

In addition to the above cognitive assessments, participants were also asked to complete questionnaires on a wide variety of other characteristics which might influence observed effects of online cognitive training. These included measures of personality (HEXACO; [77]), need for cognition [78], ‘grit’ (i.e. commitment to long term goals; [79]), and current cognitive activities [80]. Participants in the WM training and PS active control groups were also asked to complete training-specific measures of motivation to complete training, and expectations of cognitive improvement as a result of training. Measuring motivation and expected benefits of training is particularly important given the literature regarding the potential for motivational factors to artificially facilitate training effects (see [81–82]). Participants in the no-contact control group did not complete any training, and were thus not assessed for motivation of expectancy effects. All questionnaires were administered once at the beginning of the study, with the exception of the motivation and expectancy questionnaire which was administered three times: before, mid-way through, and after training.

### Training tasks

#### Working memory training program

Participants randomly assigned to the WM training group completed their online training with three games selected from Lumosity’s broader game library [68] which specifically target WM processes: 1) ‘Memory Match’ is a visual 2-back task which presents participants with an array of shapes progressing from right to left across the screen, advancing one position per trial. As the line of randomly ordered shapes progresses across the screen, it passes through two location indicator boxes two positions apart (i.e. one space between them). On each trial, participants are asked to indicate via button press whether the stimuli in the rightmost box matches that in the leftmost box which contains the stimuli from the rightmost box from two trials previous. This would be a simple matching task except that the shapes to the left of the first indicator box become invisible after several correct responses. This taxes memory for which shape was presented two trials previously, and requires continuous updating of the presented sequence. If participants respond incorrectly, all shapes in the sequence become visible until several subsequent correct responses render these shapes invisible again. 2) ‘Memory Match Overload’ is structured similarly to Memory Match, but leaves two spaces between position indicator boxes, thereby making it a more difficult visual 3-back memory task. 3) Finally, ‘Memory Lane’ mimics the logic and cognitive challenge of the dual n-back task. Participants are guided down a virtual street in which each apartment building they pass acts as one trial of the dual n-back task. At each apartment, a human silhouette appears in one of the windows and auditorily presents a letter of the alphabet. Participants are instructed to indicate via button press if either or both the location of the silhouette in the window, and auditorily presented letter, are the same as *n* apartments ago. Unlike the previous two training tasks, Memory Lane is adaptive in that the difficulty is increased when participants are successfully completing the task, and decreased when they are not, thereby ‘adapting’ to their skill level. The size of the visual stimuli presentation area (i.e. number of windows per apartment; 2x2 to 3x3), target *n* are (i.e. number of apartments ago to remember; 1-back to 10-back), and stimuli modality (i.e. visual only vs. both visual and audio) are adjusted accordingly. Game durations are 180 seconds (consisting of three 60 second rounds) for the dual n-back game (Memory Lane), 45 seconds for the 2-back game (Memory Match), and 45 seconds for the 3-back game (Memory Match overload). Each training session consisted of six Memory Match games, five Memory Match Overload games, and five Memory Lane games for a total training session time of approximately 24.5 minutes. Game order was randomized with each session and consistent between participants. Participants were asked to complete one training session per day, on five days per week, for six weeks.

#### Processing speed training program

Participants randomly assigned to the processing speed active control training group completed three different games from Lumosity’s broader game library [68] that are heavily dependent on processing speed abilities: 1) ‘Speed Match’ is a speeded visual 1-back task. It sequentially presents a series of shapes, and asks participants to quickly indicate via button press whether or not the present shape matches the one presented immediately before it. While this is a relatively simple task, emphasis is placed on improving speed of responding over the course of training. 2) ‘Speed Match Overdrive’ shares a similar structure to Speed Match, but includes a third response option for the currently presented shape to be a ‘partial’ match to that presented directly before it (e.g. matches in colour but not shape, or matches in shape but not colour). Finally, 3) ‘Spatial Speed Match’ shares the same structure as Speed Match, but includes stimuli differing only in spatial orientation. For example, two empty dots and one filled dot might be shown followed by a similar arrangement with the filled dot in a different location. Importantly, these processing speed tasks were not directly adaptive in the way that the dual n-back training was made more or less difficult by altering variables of the game. However, there was an emphasis on constant improvement through reduction of reaction times over the course of training. The three speed games (Speed Match, Speed Match Overdrive, Spatial Speed Match) last 45 seconds each and were presented 11 times per training session for a total of approximately 24.75 minutes of training per session. Consistent with the WM training group, game order was randomized with each session and consistent between participants, and participants were asked to complete one training session per day, on five days per week, for six weeks.

### Data analysis

Potential differences between the three groups before training were investigated with one-way ANOVAs, chi-squared tests, or independent samples t-tests when comparing data pertaining only to the two active training groups. To determine whether training had precipitated significant changes in cognitive test scores, a mixed-design repeated measures ANOVA was undertaken, examining time (within-subjects; before training versus after training) × group assignment (between-subjects; WM training versus PS active control versus no-contact control group) for each of the cognitive tests in our pre- and post-training test battery. For all administered subtests of the WAIS-IV, scores were converted to age-appropriate scaled scores, in order to calculate composite indices for verbal comprehension, perceptual reasoning, working memory, processing speed (VCI, PRI, WMI, PSI), as well as full-scale intelligence (FSIQ).

In addition to this traditional null hypothesis significance testing, Bayesian factors were calculated for each cognitive test via JZS Bayesian repeated measures ANOVAs in JASP version 0.8.0.0 for Windows [83–84]. JASP allows for the calculation of Bayes factors for a variety of different models, including the null hypothesis, each main factor individually (e.g. time or group), main factors combined (e.g. time + group), as well as the main factors combined with the interaction effect (e.g. time + group + time × group). Here we modelled each of the main factors as nuisance variables in order to include them with the null hypothesis, such that the interaction effect of interest (e.g. time × group) could be compared directly with its main explanatory rival—the null hypothesis including the main effects of time and group. Bayesian analyses, and Bayesian factors provide relative evidence of both null and alternative hypotheses, compared to the conclusions about the null hypothesis proffered by traditional null hypothesis significance testing [85–87].

## Results

### Participant demographics, cognitive characteristics, and personality variables

Participant groups were not statistically different on any variables measured pertaining to demographics and cognitive ability, including: age [*F*(2,73) = 0.10, *p* = .90]; distribution of males and females [χ^2^(2, N = 76) = 0.06, *p* = .97]; years of education [*F*(2,72) = 1.17, *p* = .32]; estimated full-scale intelligence quotient [*F*(2,73) = 0.70, *p* = .50], RAPM performance [*F*(2,73) = 2.25, *p* = .11]; CCFT performance [*F*(2,73) = 1.62, *p* = .21]; AOSPAN performance [*F*(2,72) = 0.28, *p* = .76]; and SDRT performance for both maintenance [*F*(2,72) = 2.32, *p* = .11] and maintenance plus manipulation [*F*(2,72) = 1.85, *p* = .17] conditions. Groups were also not statistically different on scales measuring personality characteristics, including: the Grit scale [*F*(2,69) = 0.62, *p* = .54]; the Need for Cognition scale [*F*(2,70) = 0.52, *p* = .60]; the Cognitive Activities Questionnaire [*F*(2,65) = 2.22, *p* = .12], and all dimensions of the HEXACO personality inventory. The two training groups were also equal in terms of their self-rated motivation to complete training [*t*_47_ = -0.39, *p* = .70], and their expectations of improvement on the training tasks themselves [*t*_47_ = 0.35, *p* = .73]. Table 1 summarizes these results.

**Table 1:** Participant characteristics.

	Working Memory Training Group	Processing Speed Control Group	No-Contact Control Group	Statistics
*Demographics*				
N	25	24	27	
Age	30.68 (6.24)	31.33 (5.78)	31.32 (5.58)	*F*(2,73) = 0.10, *p* = .90
Gender (male/female)	11/14	10/14	11/16	χ*2*(2, *N* = 76) = 0.06, *p* = .97
Education (years)	15.24 (2.19)	15.57 (1.93)	16.07 (1.84)	*F*(2,72) = 1.17, *p* = .32
*Cognitive Ability Before Training*				
WAIS-IV FSIQ	108.24 (15.93)	111.63 (12.34)	107.07 (13.81)	*F*(2,73) = 0.70, *p* = .50
RAPM	12.60 (3.01)	13.50 (2.27)	11.89 (2.76)	*F*(2,73) = 2.25, *p* = .11
CCFT	26.68 (4.43)	28.46 (3.51)	26.41 (4.93)	*F*(2,73) = 1.62, *p* = .21
AOSPAN total	52.79 (14.45)	53.96 (12.69)	50.93 (16.40)	*F*(2,72) = 0.28, *p* = .76
SDRT Maintenance	17.56 (1.29)	18.25 (1.39)	17.35 (1.85)	*F*(2,72) = 2.32, *p* = .11
SDRT Manipulation	16.04 (2.21)	16.63 (2.10)	15.38 (2.50)	*F*(2,72) = 1.85, *p* = .17
*Personality Factors*				
Grit score	3.44 (0.69)	3.31 (0.61)	3.44 (0.45)	*F*(2,70) = 0.36, *p* = .70
Need for Cognition score	69.36 (6.76)	69.06 (9.51)	66.92 (10.60)	*F*(2,70) = 0.52, *p* = .60
Cognitive Activities (hours/year)	1203.80 (890.50)	1407.52 (1164.60)	816.28 (788.30)	*F*(2,65) = 2.22, *p* = .12
HEXACO Honesty- Humility	3.74 (0.50)	3.80 (0.54)	3.60 (0.60)	*F*(2,70) = 0.92, *p* = .40
HEXACO Emotionality	3.00 (0.66)	2.83 (0.80)	3.18 (0.60)	*F*(2,70) = 1.53, *p* = .22
HEXACO Extraversion	3.37 (0.53)	3.54 (0.76)	3.23 (0.59)	*F*(2,70) = 1.42, *p* = .25
HEXACO Agreeableness	3.18 (0.54)	3.37 (0.50)	3.20 (0.43)	*F*(2,70) = 1.03, *p* = .36
HEXACO Conscientiousness	3.84 (0.51)	3.53 (0.64)	3.68 (0.48)	*F*(2,70) = 1.89, *p* = .16
HEXACO Openness to Experience	3.62 (0.67)	3.93 (0.42)	3.62 (0.63)	*F*(2,70) = 2.23, *p* = .12
*Training Data*				
Total hours of training	13.49 (4.86)	11.69 (3.03)	-	*t*_47_ = 1.55, *p* = .13
Pre-training motivation	5.68 (0.87)	5.77 (0.78)	-	*t*_47_ = -0.39, *p* = .70
Pre-training expectation of improvement	4.52 (0.96)	4.56 (1.10)	-	*t*_47_ = 0.35, *p* = .73

### Behavioural results

#### Training task performance and reaction Time

**Fig 2:**
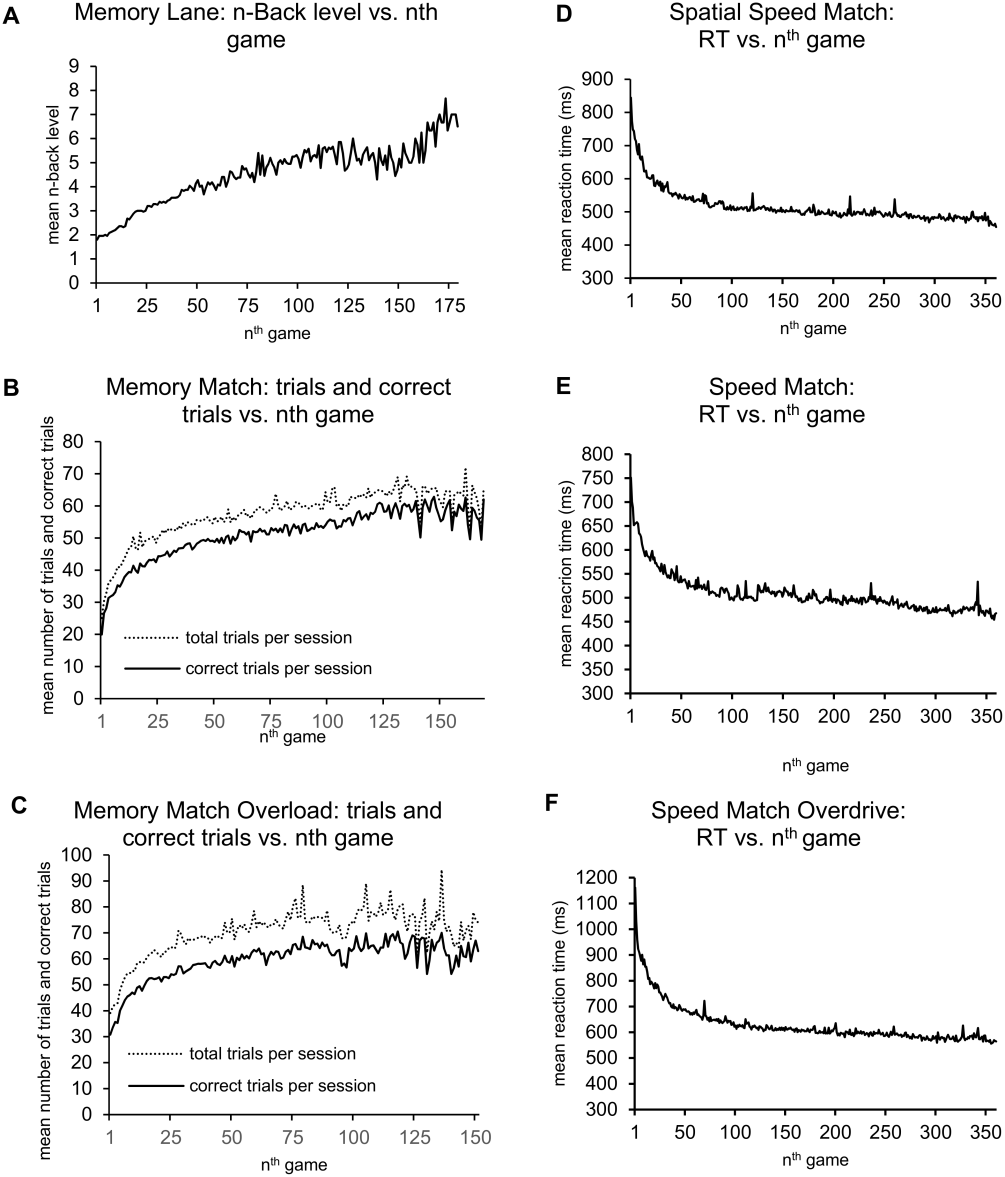
Mean performance for training tasks. Performance accuracy for the working memory training group (A-C), and mean reaction times by training game for the processing speed training group (D-F).

Members of both the WM training group and the PS active control group showed improvement on their assigned training measures across the training period. Training progress was measured for each training game by calculating a difference score between their performance on their first game, and an average of their last five games. Participants in the WM training group achieved an average *n* of 1.80 (*SD* = 0.41) on their first attempt of the Memory Lane game, and progressed to an average *n* of 5.47 (*SD* = 2.12) across their last five games, yielding a significant average difference score of 3.67 (*SD* = 2.04), *t*(24) = 9.01, *p* < .001. Additionally, WM training participants demonstrated increased proficiency on both the Memory Match, and Memory Match Overload games as indicated quantitatively by a greater number of correct matches across their last five games, compared to their first game. Difference scores were significant for both Memory Match *t*(24) = 22.63, *p* < .001, and Memory Match Overload *t*(24) = 12.41, *p* < .001. Participants also attempted a greater number of trials for these matching tasks over the course of training indicating quicker reaction times, and thereby being able to fit in a greater number of trials in a given 20-minute training session. Training progress in the PS active control group was indicated by significant decreases in reaction time across the training period. On average, participants reduced their reaction times by 367.63ms (*SD* = 233.11) on Spatial Speed Match, 278.50ms (*SD* = 142.27) on Speed Match, and 589.62ms (*SD* = 228.14) on Speed Match Overdrive when comparing their first game to the average of their last five games. Difference scores indicated significant reductions in reaction time for each of these *t*(23) = 7.73–12.66, *p* < .001. These training results are displayed graphically in Fig 2.

Importantly, the training groups were observed to have spent a statistically equivalent amount of time training with their respective online training programs over the course of the roughly six week training period: 13.69 hours for the WM training group (*SD* = 4.86), and 11.69 hours (*SD* = 3.03) for the PS active control group; *t*(47) = 1.55, *p* = .13.

#### Motivation to train and expectations for improvement

**Fig 3:**
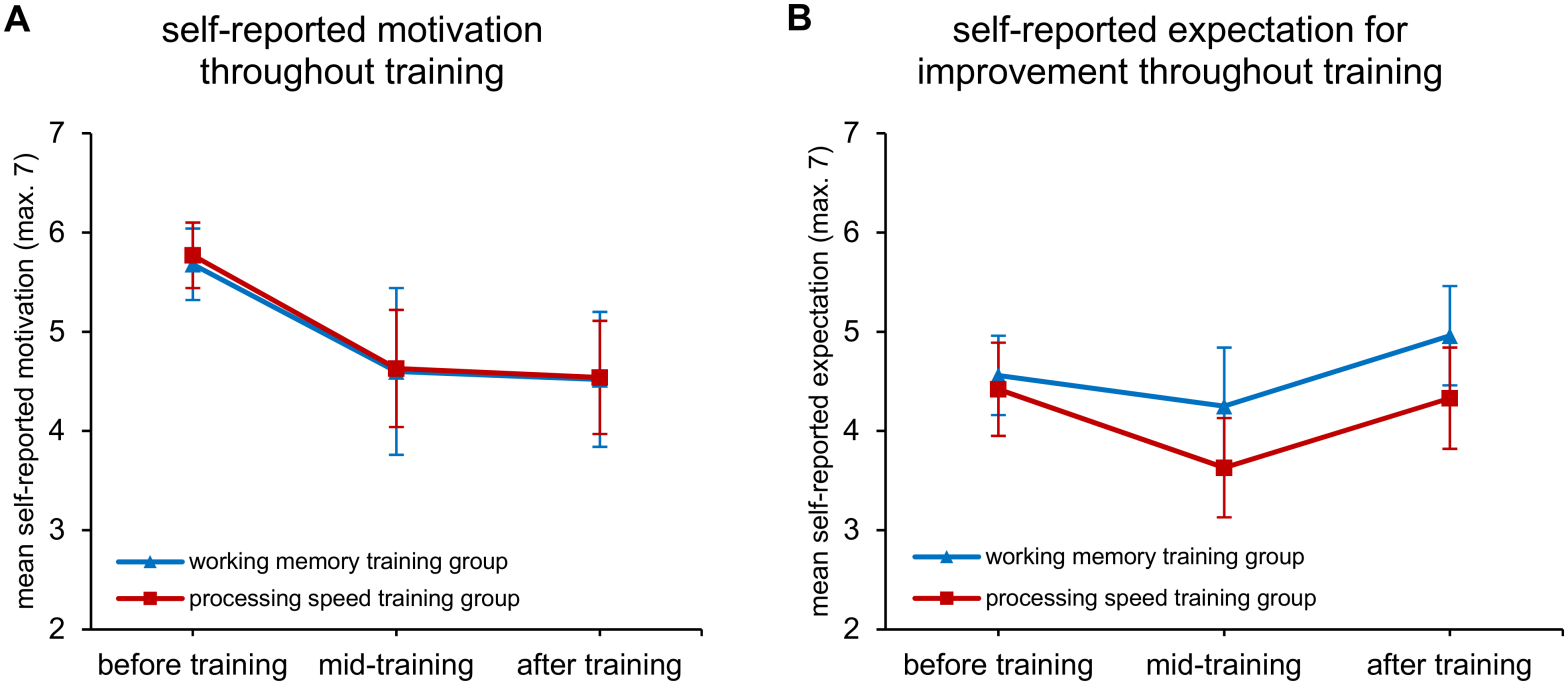
Participant motivation and expectation. Self-rated motivation to complete training (A), and expectations for improvement (B) on the training tasks throughout the training period. Error bars represent 95% confidence intervals.

Analysis of participants’ self-reported motivation to complete online training, as well as the degree to which they thought they might improve on the training tasks over the course of the training period did not reveal any significant time × group interactions. Results of the mixed-design repeated measures ANOVAs indicated main effects of time for both motivation to complete training [*F*(2,84) = 19.40, *p* < .001], and expectations for improvement [*F*(2,84) = 5.83, *p* = .004]. Bayesian analyses were carried out on these measures as well, and indicated strong evidence against the interaction effect of time × group: BF_01_ = 7.70 for motivation to complete training, and BF_01_ = 6.30 for expectation for improvement. Thus, participants in both the WM training group and PS active control group indicated a decline in motivation across the training period, but not at significantly different rates. Self-reported ratings of expectations for improvement followed a U-shaped curve for both groups, with lowest expectations for improvement mid-way through training. Fig 3 displays these metrics across the training period.

#### Cognitive test scores before and after training

**Fig 4:**
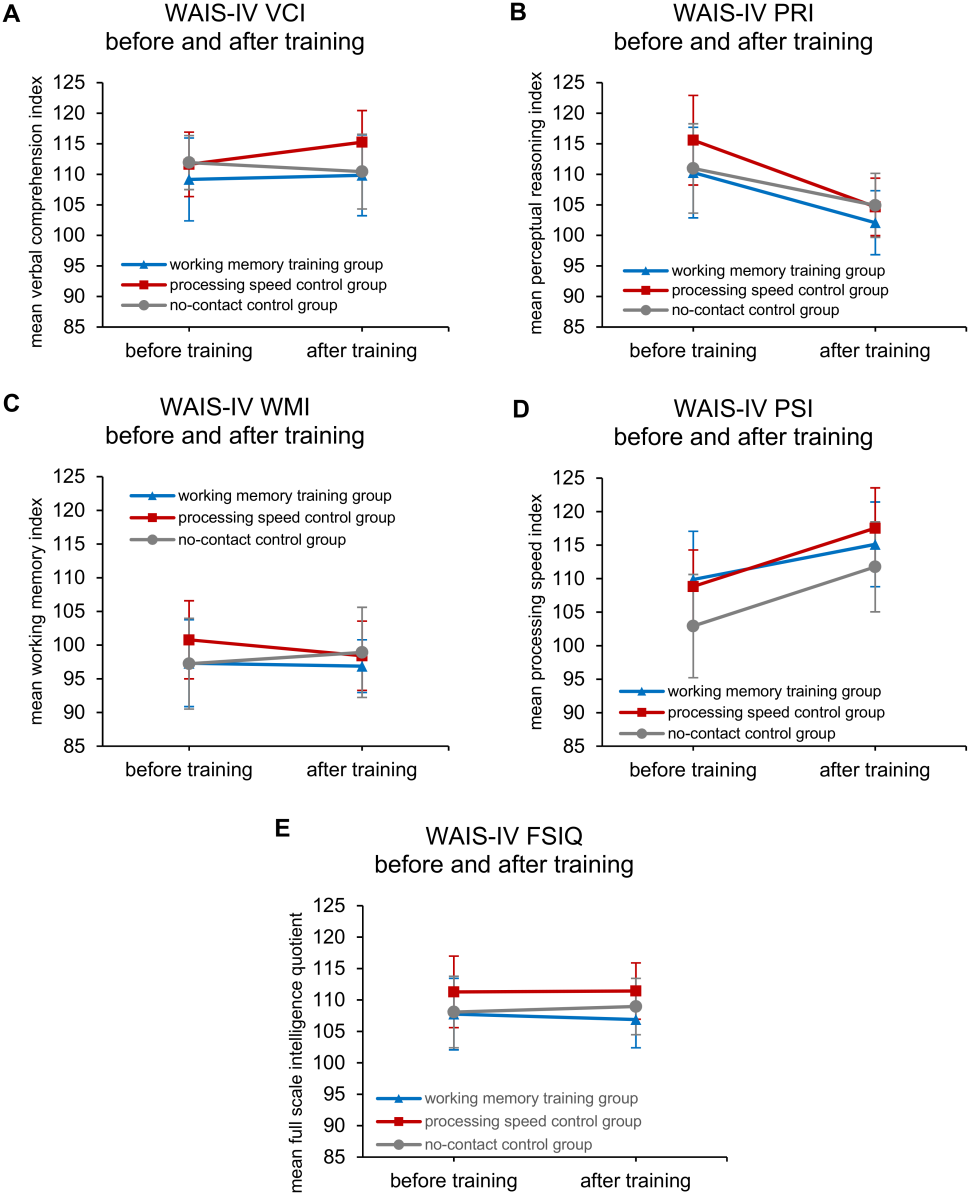
WAIS-IV performance by group, before and after training. Verbal Comprehension Index (A); Perceptual Reasoning Index (B); Working Memory Index (C); Processing Speed Index (D); Full Scale Intelligence Quotient (E). Error bars represent 95% confidence intervals.

**Fig 5:**
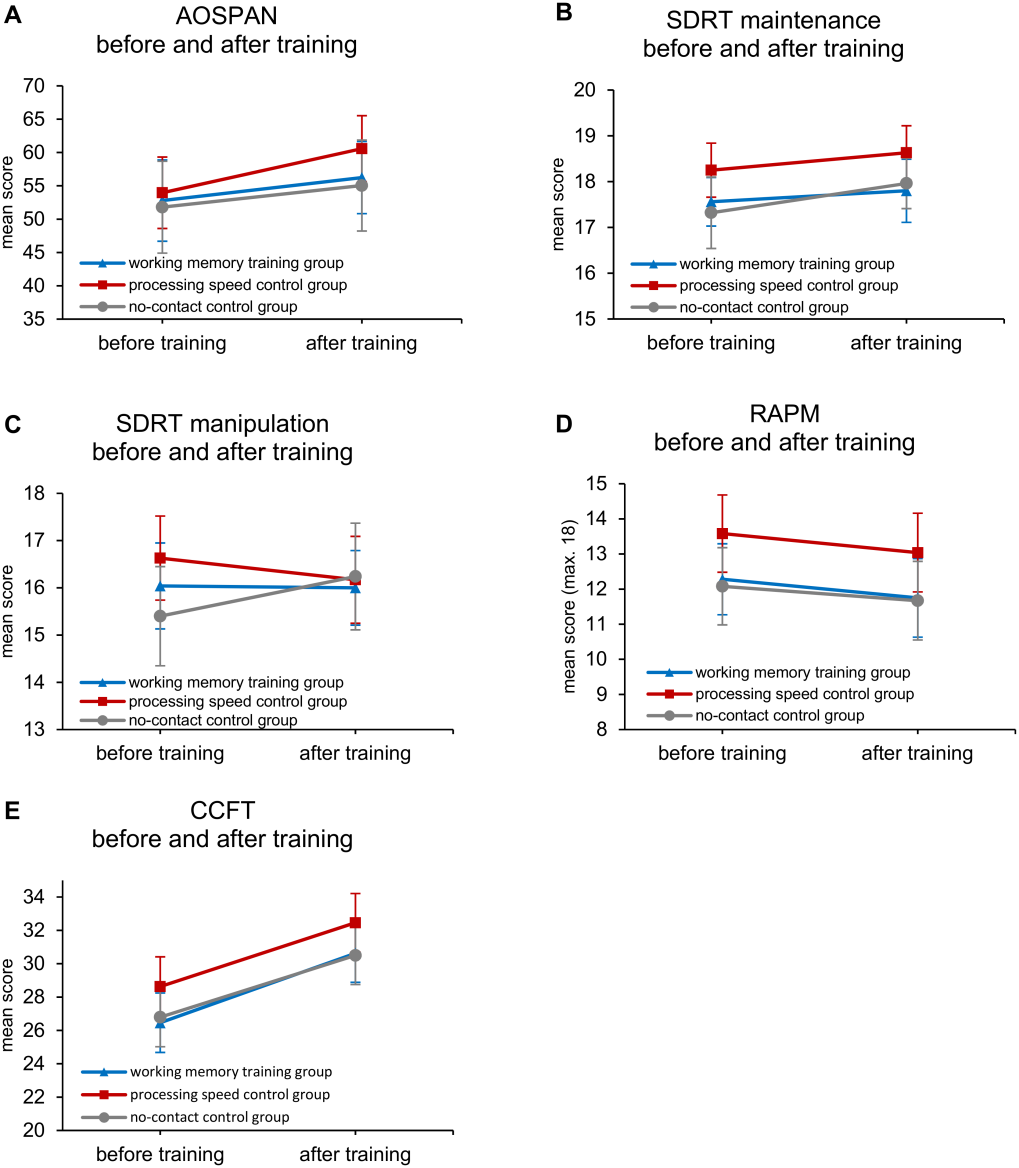
Measures of working memory capacity and fluid intelligence before and after training. Automated Operation Span (A), Spatial Delayed Response Task (B-C), Raven’s Advanced Progressive Matrices (D), and Cattell’s Culture Fair Test (E) performance by group before and after training. Error bars represent 95% confidence intervals.

Results of the mixed-design repeated measures ANOVA examining time × group for performance on cognitive testing revealed significant main effects of time for two age-normed indices of the WAIS-IV including PRI [*F*(1,73) = 24.41, *p* < .001], PSI [*F*(1,73) = 31.16, *p* < .001] (see Fig 4), as well as the AOSPAN task [*F*(1,72) = 11.85, *p* = .001], RAPM [*F*(1,73) = 4.86, *p* = .031], and CCFT [*F*(1,73) = 102.22, *p* < .001]. When raw scores from WAIS-IV subtests were used rather than age-normed composite index scores, main effects of time were found for Vocabulary [*F*(1, 73) = 13.41, *p* < .001], Similarities [*F*(1,73) = 6.57, *p* = .012], Block Design [*F*(1,73) = 37.70, *p* < .001], Symbol Search [*F*(1,73) = 12.16, *p* = 0.001], and Coding [*F*(1,73) = 31.35, *p* < .001]. Additionally, the repeated measures ANOVA revealed main effects of group membership only for the SDRT spatial maintenance task [*F*(2,72) = 3.96, *p* = .023], though very nearly for RAPM [*F*(2,73) = 3.09, *p* = .051], and the Coding subtest of the WAIS-IV [*F*(2,73) = 2.99, *p* = .057]. Follow-up pairwise analyses using the Bonferroni correction revealed a significant difference only between the PS active control group (higher scores), and the no-contact control group (lower scores) for the SDRT maintenance task. This finding is corroborated by visual inspection of the obtained data for the SDRT maintenance task (see Fig 5 panel B). In contrast to these few main effects, none of the cognitive tests administered revealed a time × group interaction effect which would be expected under the hypothesis of differential cognitive test score change by group. S1 Table displays Hedges’ *g* effect size estimates for all transfer tasks.

Further analyses with JZS Bayesian repeated measures ANOVAs were largely consistent with the results of these traditional null hypothesis significance tests. Bayes factors comparing the fit of the data under models containing the interaction term (i.e. time × group) versus the model containing only main effects by themselves (i.e. time + group) consistently indicated evidence against the interaction effect for each of the cognitive indices and subtests discussed above. Specific Bayes factors ranged from 1.06 to 9.09, indicating that the observed data are that many times more likely to occur under a model without the interaction effect, versus one that does include it. Bayes factors between 3 and 10 are thought to provide ‘substantial’ [88], or ‘positive’ [89] evidence against the interaction effect, which describes the pattern of evidence for all but two of the cognitive tests in this case: WAIS-IV Vocabulary subtest (BF_01_ = 1.06), and SDRT spatial maintenance and manipulation (BF_01_ = 2.45). These Bayes factors below 3.0 are thought to offer ‘anecdotal’ [88] or ‘weak’ [89] evidence against the interaction effect. Further, inspection of the descriptive statistics for these latter two cognitive tests for which evidence against the interaction is weakest revealed patterns of score change antithetical to gains resulting from training. These include differential *decreases* in test scores between groups over the training period for WAIS-IV vocabulary, and increases in the no-contact control group scores for the SDRT spatial maintenance and manipulation task. A table of all Bayes factor results can be found in S2 Table.

Thus, these results suggest that while participants showed facilitation of performance at the second administration after training on some cognitive tasks, none of these effects were observed to significantly vary by group.

#### Training time correlations

Interestingly, despite overall non-significant findings concerning time × group interaction effects for cognitive test scores, correlation analysis of total time spent training reveals differences between groups, and potential individual differences within the WM training group. Specifically, the total amount of time members of the WM training group spent training was significantly correlated with gains in measured WAIS-IV FSIQ (*r* = .42, *p* = .039), however, not for any of the constituent composite indices (VCI, PRI, WMI, PSI; *r*’s = .13–.38, *p*’s = .06.28), nor intermediate measures of working memory ability (AOSPAN task and SDRT; *r*’s = -.31–.31, *p*’s = .14–.89), nor either measure of nonverbal ability administered (RAPM, CCFT; *r*’s = -.07–.06, *p*’s = .75–.76). Conversely, total time spent training by members of the PS active control group was not found to be significantly associated with observed gains in FSIQ, nor any of the above listed measures (*r*’s = -.36–.33, *p*’s = .08–.81) with the one exception of VCI (*r* = .42, *p* = .039).

## Discussion

The goal of the present study was to evaluate the weight of evidence for or against the controversial claim that WM training ‘works’; or more specifically that training of WM transfers to untrained cognitive tasks in the domain of fluid intelligence. We evaluated this hypothesis in a community-recruited sample of healthy young adults, aged 18–40, in a randomized controlled six week trial of online WM training compared to both active and no-contact control groups.

The present results provide no convincing evidence of near-transfer of WM training to WM capacity, or far-transfer to *Gf* despite significant improvement on all training tasks across both groups. Similarly, improved performance on the WM or PS training tasks did not demonstrate far-transfer to a broad range of cognitive domains measured by a traditional comprehensive test of intelligence. Stated plainly, participants randomized to six weeks of online working memory training fared no better on these cognitive tasks after training, when compared to those randomized to a processing speed active control condition, or even compared to those randomized to a no-contact control condition. Several cognitive tests and indices evinced higher scores at the post-training cognitive assessment relative to the pre-training assessment (e.g. WAIS-IV PRI, PSI; AOSPAN; RAPM; CCFT); however, in each case, the effect did not significantly differ by group, suggesting practice effects for the tests themselves versus true training-related gains in performance [90]. Overall, this pattern of results supports our first hypothesis (that participants would improve on training tasks), though provides substantial evidence against our more consequential second hypothesis (that WM training would precipitate near-transfer to WM capacity), and third hypothesis (that WM training would precipitate far-transfer to *Gf*).

Counter to these results, post-hoc analyses revealed that total time spent training by members of the WM training group was positively and significantly correlated in observed gains in overall intelligence as measured by the WAIS-IV full-scale intelligence quotient (FSIQ) index. This pattern did not obtain for the PS active control condition. However, two indicators suggest that this finding should be interpreted with caution, if not completely disregarded. First, similar correlations did not hold for constituent indices of the FSIQ (i.e. VCI, PRI, WMI, or PSI). Second, total time spent training by members of the PS active control group was positively and significantly correlated with gains on WAIS-IV VCI (composed of tests of vocabulary and abstract verbal reasoning) for which there is no theoretical basis for improvement following training of processing speed. Rather, both of these correlations are more than likely spurious, resulting from measurement error and/or psychometric imprecision (discussed below).

Looking to the literature, these results are consistent with a large and growing body of empirical work in support of the null for WM training [see 26, 36–45]. However, due to the largely divided or ‘reliably ambiguous’ [60] nature of the current WM training literature, these results are also inconsistent with a large and growing opposing body of empirical work that has demonstrated evidence for both near- and far-transfer effects resulting from WM training in healthy adults [28–35, 43].

While the present results land firmly and unambiguously on the former side of this split literature, the addition of our single empirical result cannot hope to ultimately settle the debate on WM training efficacy. However, a more targeted comparison of study methodology may provide several clues as to *why* it found support for the null. For example, following Melby-Lervåg and colleagues [53] analysis, narrowing the broader WM training literature to only the 34 comparisons to date which have included 20 or greater participants per group, and also utilized an active control condition revealed a negligible mean effect size. In comparison, every other combination of experimental design (e.g. < 20 participants per group, with untreated controls etc.) yielded significant mean effect sizes. In other words, the literature composed of methodologically rigorous studies is not so split or divided as the broader WM training literature, and the present results are indeed consistent with these similarly rigorous experimental trials.

Despite methodological rigor on these important points, limitations of the current study include equivalence of pre- and post-training cognitive test forms, as well as a high degree of participant attrition from the both the WM training group and the PS active control group. First, regarding the equivalence of test forms, here we split singular tests into roughly equivalent versions according to even and odd item numbers. However, because most of these cognitive tests are designed such that each successive question is incrementally more difficult than the last, it remains possible that the form containing even-numbered items is slightly more difficult than the one containing odd-numbered items despite good psychometric properties in terms of split-half reliabilities. In the present experimental design, we decided on the most conservative option which is to use the odd-numbered items at pre-training assessment, and even-numbered items at post-training assessments.

Regarding participant attrition, it should be noted here that while only 7 and 8 participants withdrew from the WM training and PS active control conditions (or abandoned their prescribed training plan) respectively, these numbers represent a rather large proportion of the total group sizes (7/32 = 21.89% for the WM group, and 8/32 = 25% for the PS group). This drop-out may speak to any number of factors about the tolerability of the interventions, and leaves the current results open to speculation about potential systematic differences between trial completers and trial abandoners. Anecdotally, several participants noted in conversation with the experimenters that training became less exciting and somewhat repetitive across the six week training period. These sentiments are corroborated quantitatively for both the WM training group and PS active control group by substantial decreases in self-rated motivation and expectations of improvement from training between the start of the trial and even halfway through. Several participants (from both the WM and PS groups) expressed that adding more variety to the training regimen may have served to enhance its appeal. Regardless of whether the repetitive nature of the highly circumscribed sets of training tasks accounts for any of the participant drop-out, Straus, Glasziou, Richardson & Haynes [91] discuss the implications of attrition from RCTs, and point out that many medical journals will refuse to publish trials with attrition rates above 20%. Examination of the factors that lead to WM training adherence and attrition will be important topics of future research (see [92]). Post-hoc analyses revealed few statistically significant differences between cognitive and questionnaire baseline characteristics of participants who abandoned the study after randomization, and those who completed the trial. Specifically, those who dropped out of the study were found to have higher scores on the AOSPAN task, and lower scores on the ‘fearfulness’ facet of the HEXACO personality inventory. Importantly however neither of these significant results survive the Bonferonni correction for multiple comparisons (i.e. ~50 separate t-tests).

Finally, while the current study includes just over the minimum number of 20 participants per group recommended by the literature [62], it should be pointed out that power analyses based on an early estimate of a moderate mean effect size of *d* = 0.34 for n-back training studies [56] would require samples sizes of 108 participants per group in order to achieve a power of 0.8 with an α = .05 in a 1-tailed independent samples t-test with equal sample sizes. Given group sizes of 24, the power of the present study sits at roughly 0.3. The danger here of course is that low values for statistical power such as this lead to poorer chances of detecting an effect if it truly exists, and also poorer chances that any found effects are indeed genuine [93–95]. Thus, regardless of minimum participant number suggestions from the literature, this power analysis indicates a meager ~30% chance of the present study finding a moderate effect of WM training if it actually exists. Future research on WM training efficacy will benefit from greater statistical power resulting from larger sample sizes. Online tools for homogenizing study design and streamlining participant training will certainly aid in organizing larger multi-site WM training studies (see [96] for an early example).

These limitations notwithstanding, our trial includes several strengths that work to improve upon methodological shortcomings that have been described as ubiquitous or pervasive in the existing WM training literature [53, 66, 63, 95]. In addition to utilizing minimum suggested sample sizes, and employing an active control condition, the present study sought to reduce the ambiguity of potential positive findings by measuring a number of intra-individual variables that have been suggested to moderate WM training effect, including: self-rated motivation to complete training, self-rated expectations of cognitive improvement from training, major personality traits, grit, need for cognition, as well as current cognitive activities. By measuring and ensuring equivalence between groups on these potentially important intra-personal variables, in addition to vital demographic characteristics (i.e. age, sex, education, and IQ), their impact on any potential gains in cognitive ability can be effectively ruled out. No such gains in ability were observed in this case, however because these traits were measured, we can state with some confidence that our null findings were not due to unmeasured differences in these variables between our three groups. The near-perfect equivalence of our three groups on all of the above variables precludes the necessity to statistically model pre-training group differences in our analyses. Additionally, and contrary to much of the previous literature, we utilized multiple measures of the cognitive domains of interest: working memory (WAIS-IV Digit Span, and Arithmetic, AOSPAN, SDRT), and fluid intelligence (WAIS-IV Matrix Reasoning, and Block Design, RAPM, and CCFT which is composed of four separate tests of *Gf* ability). Each of these measures within these given domains of interest returned consistent results in support of the null regarding WM training.

A final strength of our trial is that cognitive test scores were not observed to decrease over the course of the training period for either of the control groups, which Redick [66] has pointed out as a contributing factor to significant time × group interactions in several successful WM training studies. It is interesting to point out however, that while including an active control condition that closely matches all but the proposed intervention of the treatment group is certainly a methodological asset, our active and passive control groups obtained very much the same result–i.e. no significant improvement on any cognitive test which could not otherwise be due to expected practice effects. This is an interesting and somewhat unexpected result given the large discrepancies in average mean effect sizes listed in meta-analytic reviews. Recall that Melby-Lervåg and colleagues [53] found effect sizes of 0.15 and 0.26 for n-back training on nonverbal ability for treated and untreated controls, whereas Au and colleagues [55] found an even larger discrepancy with effect sizes of 0.06 and 0.44 for treated versus untreated controls in their more targeted review. Heterogeneity of study design in the WM training literature makes it difficult to compare the equivalence of our active and passive control conditions to previous studies. An in depth examination of Melby-Lervåg and colleagues [53] supplementary material yielded no comparable studies meeting the following criteria: 1) sample of young adults (vs. children or older adults); 2) 20 or greater participants per group; 3) participants randomized to both active and passive control groups in addition to the treatment group(s); 4) utilization of the dual n-back task for training; 5) examination of fluid intelligence as an outcome measure. The closest experimental trial to these criteria is that of Redick et al. [40], which meets all of the above conditions except true group randomization. Interestingly, their results indicated a similar pattern to those found here: non-significant differences between all three groups, including both active and passive control conditions. These results raise the thorny question of whether other trial- or researcher-specific factors may account for some of the variance observed between studies which include active control conditions, and those that do not (e.g. experimenter bias, publication bias etc.). Notably, Redick et al.’s [40] trial also shares in common with the current study, the failure to find near-transfer of training to measures of WM span, or WM capacity, contrary to many findings to this effect in the literature [28, 49–50].

In sum, the present study found no convincing evidence of far-transfer of WM training to untrained measures of *Gf*, nor near-transfer of training to intermediate cognitive domains (i.e. WM capacity) thought to mediate increases of *Gf* in young adults. Importantly, we implemented a methodologically rigorous design following recommendations from recent literature, and also measured a variety of intra-personal factors that have been proposed to moderate treatment effect. Overall, while the present results in support of the null cannot hope to singly resolve the heated debate over the controversial claims of WM training efficacy, they do contribute meaningfully to the rapidly growing corpus of research on the topic. Crucially, by providing additional and incremental evidence against the efficacy of dual n-back training in healthy young adults, subsequent research can intensify the search for alternative interventions that may produce the desired effects in this population (see [97]), or alternative populations or patient groups for which dual n-back training may actually be effective (see [98] for a review, and [57] for a meta-analysis).

## Supporting information

S1 Table Pre- to post-training effect sizes for transfer tasks following Melby-Lervåg & Hulme (2016), and Morris (2008). (DOCX)

S2 Table Bayesian factors for time × group interactions, by measure derived from the JZS Bayesian repeated measures ANOVAs. (DOCX)

S1 Fig Recruitment poster. (TIF)
