# Supplementary material for: Working memory training in healthy young adults: Support for the null from a randomized comparison to active and passive control groups
Source: PLoS One. 2017 May 30;12(5):e0177707. doi: 10.1371/journal.pone.0177707 (PMC5448748; doi:10.1371/journal.pone.0177707)
Supplement: S1 Table — (DOCX) [file pone.0177707.s001.docx]

S1 Table.

*Pre- to post-training effect sizes for transfer tasks following Melby-Lervag & Hulme (2016), and Morris (2008)*

| Cognitive Measure | Effect Size (Hedges’ g) |
| --- | --- |
| Raven’s Standard Progressive Matrices | -0.081 |
| Cattell’s Culture Fair Test (total score) | 0.110 |
| Spatial Delayed Response Task (maintenance) | -0.103 |
| Spatial Delayed Response Task (manipulation) | 0.192 |
| Automated Operation Span Task (total score) | -0.229 |
