# Supplementary material for: Working memory training in healthy young adults: Support for the null from a randomized comparison to active and passive control groups
Source: PLoS One. 2017 May 30;12(5):e0177707. doi: 10.1371/journal.pone.0177707 (PMC5448748; doi:10.1371/journal.pone.0177707)
Supplement: S2 Table — (DOCX) [file pone.0177707.s002.docx]

S2 Table.

*Bayesian factors for time* × *group interactions by measure derived from the JZS Bayesian repeated measures ANOVAs*

|  | BF_01_ | Quality of evidence against interaction effect | |
| --- | --- | --- | --- |
|  |  | Jeffreys (1961) | Raftery (1995) |
| *WAIS-IV Composite Indices* |  |  |  |
| Full-Scale IQ | 7.51 | Substantial | Positive |
| Verbal Comprehension Index | 3.66 | Substantial | Positive |
| Perceptual Reasoning Index | 5.22 | Substantial | Positive |
| Working Memory Index | 5.90 | Substantial | Positive |
| Processing Speed Index | 4.67 | Substantial | Positive |
|  |  |  |  |
| *WAIS-IV Subtests* |  |  |  |
| Vocabulary | 1.06 | Weak | Anecdotal |
| Similarities | 8.24 | Substantial | Positive |
| Block Design | 4.88 | Substantial | Positive |
| Matrix Reasoning | 4.39 | Substantial | Positive |
| Digit Span | 3.61 | Substantial | Positive |
| Arithmetic | 8.65 | Substantial | Positive |
| Symbol Search | 4.56 | Substantial | Positive |
| Coding | 6.95 | Substantial | Positive |
|  |  |  |  |
| *Computerized Working Memory Tasks* |  |  |  |
| Automated Operation Span | 4.16 | Substantial | Positive |
| SDRT Spatial Maintenance Task | 7.7 | Substantial | Positive |
| SDRT Spatial Maintenance and Manipulation Task | 2.45 | Weak | Anecdotal |
|  |  |  |  |
| *Nonverbal Reasoning Tasks* |  |  |  |
| Raven’s Advanced Progressive Matrices | 8.51 | Substantial | Positive |
| Cattell’s Culture Fair Test | 9.09 | Substantial | Positive |
|  |  |  |  |
| *Motivation and Expectation* |  |  |  |
| Motivation to complete training | 7.70 | Substantial | Positive |
| Expectation for improvement | 6.30 | Substantial | Positive |
