# Supplementary figures and images for: Working memory training in healthy young adults: Support for the null from a randomized comparison to active and passive control groups
Source: PLoS One. 2017 May 30;12(5):e0177707. doi: 10.1371/journal.pone.0177707 (PMC5448748; doi:10.1371/journal.pone.0177707)

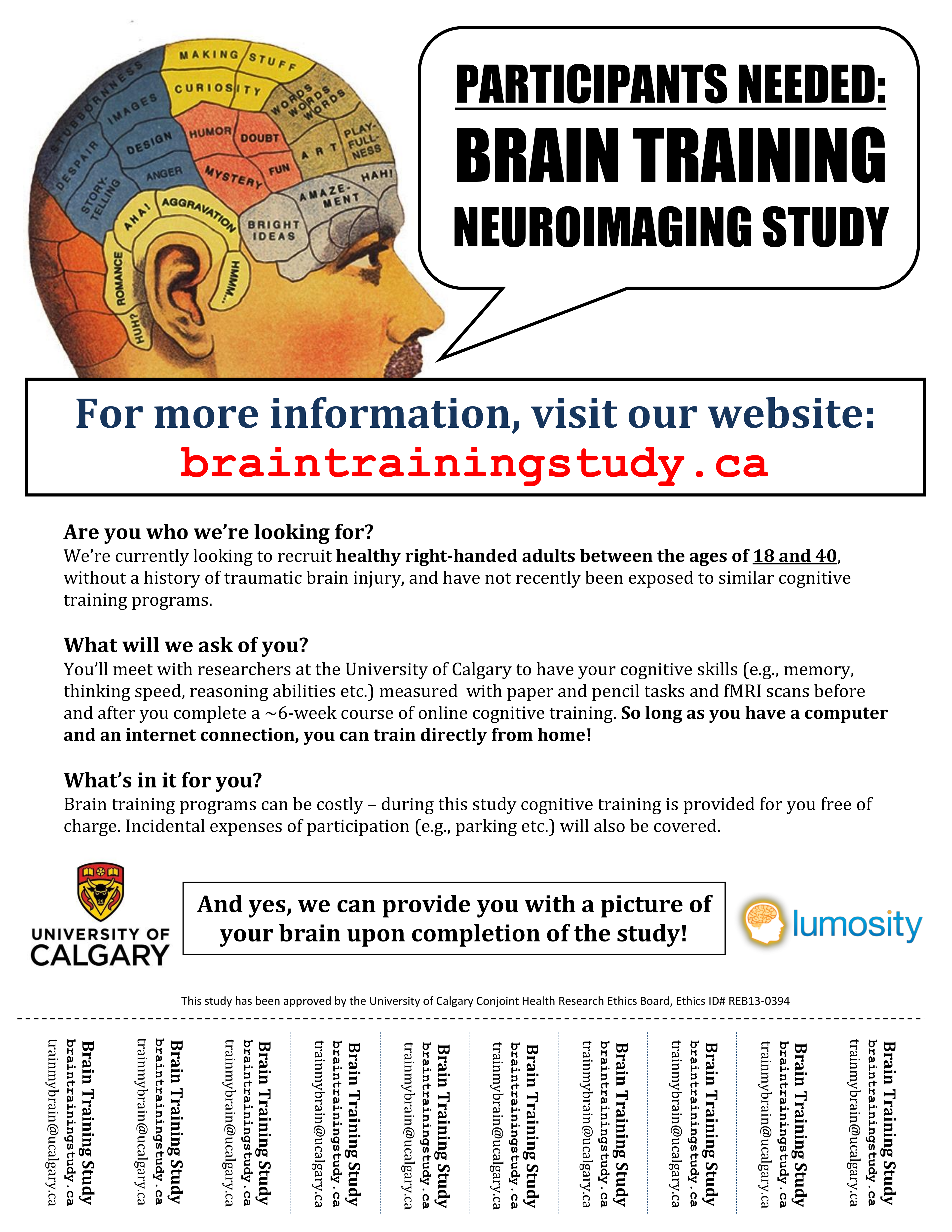

Supplement: S1 Fig — (TIF) [file pone.0177707.s003.tif]
